# The role of community health nurses in promoting school learners’ reproductive health in North West province

**DOI:** 10.4102/hsag.v28i0.2109

**Published:** 2023-03-07

**Authors:** Tshiamo N. Ramalepa

**Affiliations:** 1 Department of Nursing Science, School of Healthcare Sciences, Sefako Makgatho Health Sciences University, Tshwane, South Africa

**Keywords:** school learners, reproductive health in schools, community health nurses, reproductive health, North West province

## Abstract

**Background:**

Reproductive health education is a major component in schools, which is delivered through Life Orientation and Life Science subjects. Providing sexual and reproductive health education and services remains a challenge in schools of many countries, as well as South Africa. Community health nurses have the responsibility to initiate and participate in reproductive health promotion initiatives in schools.

**Aim:**

To explore the roles of community health nurses in the promotion of school learners’ reproductive health in schools.

**Setting:**

This study was conducted in the clinics of Madibeng municipality in North West province, South Africa.

**Methods:**

An exploratory qualitative research study was conducted using in-depth interviews for data collection. The population included community health nurses who were sampled purposively.

**Results:**

Community health nurses revealed that their primary role was to provide health education to learners, particularly in clinics. Furthermore, they revealed that they did not visit schools and had no communication with teachers regarding learners’ reproductive health issues.

**Conclusion:**

The Department of Education has opened a platform for the provision of reproductive health education in schools through various teacher-led initiatives. However, this has posed a significant challenge to teachers as they may not be willing to deliver sensitive and sexually themed information to learners. To ensure effective delivery of reproductive health education in schools, community health nurses, teachers and other relevant stakeholders must collaborate in schools.

**Contribution:**

This article highlights the importance of community health nurses visiting schools to promote the reproductive health of school learners.

## Introduction

Reproductive health is a state of complete physical, psychological and social well-being, and it is mainly related to an individual’s reproductive system (United Nations Population Fund 2021). Reproductive health is a major component in schools (Joseph et al. 2021). One reproductive health issue that commonly presents itself in schools is teenage pregnancy (Nkosi & Pretorius 2019:108). The World Health Organization (WHO) (WHO 2021) explained the intricacy of teenage pregnancy globally by stating that about 21 million teenage girls between the ages of 15 and 19 years become pregnant in developing countries. Furthermore, at least 777 000 girls under the age of 15 years give birth in developing countries (WHO 2021). As most teenagers are enrolled in primary and secondary schools, teenage pregnancies occur within the school environment. Teenage pregnancy has been highlighted as a major factor that perpetuates maternal mortality and morbidity, as well as early school dropout in sub-Saharan Africa (Barron et al. 2022:252). In the United States of America (USA), a wide range of healthcare services are provided in the school environment through school-based healthcare centres; however, there is still a challenge in providing reproductive health services in schools (Briggs et al. 2021).

For school learners to be able to maintain their reproductive health and make informed decisions, they need access to accurate information, and safe, affordable and effective preventative sexual and reproductive health services in schools (UNFPA 2021). In South Africa, much like many countries, providing reproductive health services in schools remains a challenge (Nkosi & Pretorius 2019:109). However, Matlala, Nolte and Temane (2014:85) suggested that the current school curriculum makes an effort to address reproductive health in schools although this has not had an impact in reducing the rise in teenage pregnancies.

Recent media reports indicate that the rise in teenage pregnancy rates in South Africa has been exacerbated by the COVID-19 pandemic (Molek & Bellizzi 2022:218). Between 2018 and 2019, South Africa recorded a rise in teenage pregnancy rates; moreover, because of the COVID-19 lockdown, teenage pregnancy rates increased even further (Jonas 2021). It was reported that between April 2020 and March 2021, more than 23 000 girls under the age of 18 years gave birth in Gauteng province alone (Channels Television 2021); 70% of the overall national pregnancy cases among teenagers were reported to be unplanned (Jonas 2021). The effort to curb the rise in teenage pregnancies in South African schools is addressed through reproductive health policy-led initiatives. Pregnancy in school-going children has been attributed to reproductive ignorance, early occurrence of menarche, risk-taking behaviour and psychological problems (Selebetswe, Dibakwane & Mmapheko 2018:2). Some of the contributory factors include lack of parental care, poor reproductive health education in schools, poor access to contraceptives, misuse or incorrect use of contraceptives and also issues relating to termination of pregnancy (Gwido & Fakadu 2016:28). Community nurses have more to do in improving the health of school-going children through regular visits to schools to raise awareness. Selebetswe et al. (2018:2) highlighted the challenges faced by community health nurses in visiting schools; they include shortage of staff in clinics, lack of facilities and lack of support by school management boards. These challenges may have a negative impact on school health programmes.

Whitehead (2016:265) revealed that a number of school-based initiatives have been introduced to promote the health of teenagers in schools. World Health Organization (2022) identified schools as health promotion institutions that have the capability to utilise their capacity to meet the health needs of school learners. The schools’ capacity can be improved to include the role of health professionals. More than two decades ago, the Department of Health (DOH) in South Africa emphasised that health promotion in schools should not be seen as an extra burden in schools but should be used as an effective way to address health-related issues within the school curriculum (KwaZulu-Natal Department of Health 2001). In recent years, the Department of Basic Education (DOBE) views health promotion as a tool to create healthy school environments by promoting the general health of school learners (DOBE 2021). The school environment should be regarded as an important space for health promotion initiatives, whereby health promotion interventions are integrated into the school curriculum (Whitehead 2016:264). Notable health promotion initiatives that are included in the school curriculum are the Integrated School Health Policy (ISHP), peer education programme, HIV and AIDS life skills education programme, alcohol and drug use prevention and management programme, and the care and support for teaching and learning programme (DOBE 2021). Furthermore, the involvement of community health nurses as important stakeholders in implementing such programmes is supported by the ISHP, which identifies the community health nurse as the facilitator of the policy (Mulaudzi & Peu 2014). Community health nurses play an important role in implementing health policies in the community; they carry out their roles working in community-based clinics. Conversely, community health nurses need to work hand in hand with other departments such as the DOBE and the Department of Social Development to promote health in schools. Although Mulaudzi and Peu (2014) emphasised that the policy environment describes the role of community health nurses in facilitating the implementation of healthcare services in schools, it is unclear how nurses understand their role in promoting school learners’ reproductive health. Therefore, the aim of this study was to explore the roles of community health nurses in the promotion of learner’s reproductive health in schools of Madibeng municipality, North West province.

## Methodology

### Design

This study followed a qualitative exploratory research design that was conducted in the clinics of Madibeng (Brink, Van der Walt & Van Rensburg 2018:104). This qualitative research design was used to explore the roles of community health nurses in promoting school learners’ reproductive health in schools of Madibeng, North West province.

### Setting

This study was conducted in the clinics of Madibeng municipality, North West province. Madibeng municipality is one of six municipalities in Bojalana District in North West province, South Africa. There are about 178 community health nurses who are based in clinics and community healthcare centres in Madibeng. Madibeng is known for its diversified economy, which is dominated by mining, manufacturing, tourism and agriculture (Municipalities of South Africa 2017).

### Population and sampling

The population of the study consisted of 10 community health nurses in Madibeng municipality. In this study, community health nurses referred to professional community nurses based in clinics and community healthcare centres, who were responsible for the provision of health service in the community. Participants who were considered for participation included professional community health nurses working for the Madibeng sub-district health department. Community health nurses who were not considered for participation included community health nurses of lower categories such as enrolled nurses and enrolled nursing assistants. Purposive sampling method was used to select participants, and the researcher used this sampling method to choose participants who were experienced and knowledgeable about the phenomenon of interest and had at least 2 years of working experience as community health nurses (Botma et al. 2010:201). The sample size was 10 community health nurses. Saturation was used to determine the sample size, and this was done when no new information emerged during data collection (Brink et al. 2018:126).

### Data collection

An appointment was made with clinic managers and community health nurses the day before data collection. Data were collected by means of individual in-depth interviews. An interview guide containing open-ended questions was used to facilitate the interview process. The interviews were conducted in the clinics’ boardrooms during a time when participants were not engaged with patients. The individual interviews were recorded using a tape recorder after participants were given information regarding the purpose of the study and what participation entailed. Participants had to give permission before the tape recorder was used. Each interview took approximately 30 min. Permission to conduct the study was obtained from the North West Department of Health, provincial office. The interview guide comprised one central question, followed by potential probing questions. The central question read as follows: ‘Please tell me what are your roles in promoting school learners’ reproductive health in schools of Madibeng?’

### Data analysis

Data were analysed by means of content analysis using Tesch’s approach (Polit & Beck 2012:281). To analyse the data using content analysis, all the recorded interviews were transcribed verbatim. The raw data were then cleaned and sorted, and then, the author coded the transcripts to enable the development of categories and themes. Through the data analysis process, themes that were more meaningful emerged from the coded and categorised data. An independent coder was given the audio recordings and transcripts to further analyse the data and confirm the author’s emerged themes. Furthermore, the independent coder, the researcher and the supervisor discussed the findings of the study in order to reach a consensus about the categories and the themes. The purpose of the consensus discussion was to mediate any differences and disagreements in the findings.

### Measures to ensure trustworthiness

The research findings are based on the views and experiences of participants; trustworthiness was ensured through credibility, transferability, dependability and confirmability (Stahl & King 2020:26). Credibility was ensured by prolonged engagement, where the researcher was familiar with the study because he was previously a professional nurse in one of the local clinics. The researcher is a community health nurse and was supervised by qualified supervisors who have doctorates in nursing and are knowledgeable in research. Transferability was ensured by carefully selecting participants using a purposive sampling method, with clear inclusion and exclusion criteria. Dependability was ensured by keeping records for an audit trail to enable effective auditing of the collected data and by using similar venues and settings to conduct the interviews. All the interview transcripts, audiotapes, voice recordings and field notes were kept and stored for 5 years as an audit trail. Confirmability was ensured by conducting a confirmability audit to ensure that the data collected were accurately interpreted and reflected the participants’ responses. Furthermore, all data transcripts were sent to the independent coder for further analysis, and the researcher used a literature search to confirm findings.

### Ethical considerations

In research studies, researchers must adhere to a specific ethical code of conduct that is aimed to protect research participants (Bhandari 2021). The ethical principles followed in this study include autonomy, privacy and confidentiality, informed consent, justice, non-maleficence and beneficence. Participants in this study were chosen fairly using purposive sampling; informed consent was obtained before the data were collected and all participants were free from cohesion and prejudice regarding their choice of participation in the study. Privacy was ensured by conducting the interviews in a private boardroom, and the names and personal details of the participants were not used in any report to ensure confidentiality. Ethical clearance to conduct the study was obtained from the Research Ethics Committee of Tshwane University of Technology (Reference number: REC/2018/11/005). Permission to conduct the study was obtained from the North West Department of Health, as well as the clinic managers.

## Results

The following three themes emerged from data analysis: Theme 1 was nurses’ involvement in learners’ reproductive health issues, theme 2 was the roles of clinics in learners’ reproductive health and theme 3 was stakeholders involved in learners’ reproductive health. The findings are presented as themes. Vignettes will be used as evidence to represent participants’ actual voices.

### Theme 1: Nurses’ involvement in learners’ reproductive health issues

Community health nurses suggested that their primary role was to provide health education to learners, particularly when learners visit the clinic in the reproductive health units. Although they provided health education to learners, they only did it within the vicinity of the clinic. It was also affirmed that community health nurses did not visit schools as part of their community outreach programmes. They indicated that there was no communication with teachers regarding pregnant learners’ referrals and follow-up care. Community health nurses identified health education as their main responsibility towards school learners:

‘My role as a healthcare practitioner is health education that’s where it all starts, we must give health education regarding prevention of teenage pregnancy. My other role is to offer family planning services like contraceptives….’ (Participant 1, female, community health nurse [CHN])

‘We first have to prevent pregnancy by educating them about family planning and how to access it and how we provide those services to them and manage pregnancy while they are still in school. We encourage them to book early; we give them information on how they can cope with pregnancy and antenatal visits.’ (Participant 4, female, CHN)

‘According to me is to give health education every day and even if it’s few people the information must be spread. These young learners are a big problem because by the time she comes this side she has no idea on what is supposed to be done during their check-ups we struggle a lot with them.’ (Participant 9, female, CHN)

Community health nurses indicated that they were not involved at all in school visits and that there were no outreach programmes. Health education and reproductive health services were only provided when school learners came to the clinic. The outreach programmes only focused on initiatives such as the immunisation programme:

‘Right now there is not much that I am doing because there are no longer outreaches where nurses will go out to schools and teach children and all that…there isn’t much that is going on.’ (Participant 7, female, CHN)

‘We have healthcare workers who go around, but I am not certain if they visit schools. Personally I haven’t went there, and I have never heard that there is a team of nurses visiting schools for health education unless if it is things like immunisations.’ (Participant 6, female, CHN)

Community health nurses seemed unsure about their roles in schools. However, they revealed that they were aware that they should provide health education in schools. With regard to communication with teachers about referrals and follow-up care of learners, they revealed that they had no communication with the teachers and that there was no referral system or follow-up care in place regarding learners’ reproductive health issues. Furthermore, it is the responsibility of the learner and her parents to ensure that she visits the clinic and not the responsibility of the teacher or nurse:

‘There is no communication, unless it is parents because with parents we do unlike with schools. It will be good because most of the time they are at school so they need to accommodate them so that they could come for their appointments, so that if I give a follow-up date.’ (Participant 4, female, CHN)

‘There is no communication; these learners are just coming to the clinic at their own will. I think teachers don’t care if they see children being pregnant because you will find a young child being pregnant and coming alone to the clinic.’ (Participant 1, female, CHN)

‘We actually don’t have direct contact with teachers we only communicate with parents because this minor’s parents they accompany them to the clinic.’ (Participant 5, female, CHN)

The general suggestion among community health nurses was that there should be a communication and referral system between nurses and teachers regarding school learners’ reproductive health issues.

### Theme 2: The role of the clinics in learners’ reproductive health issues

The findings suggest that clinics do not have access to policies that address learners’ health issues such as the learner pregnancy policy and the ISHP. Therefore, lack of knowledge regarding learners’ reproductive health policies was prominent. The findings revealed that clinics did not participate in school health initiatives and that community health nurses were not participating in addressing learner pregnancy in the school environment. They could only be in contact with pregnant learners when they attended antenatal classes:

‘Learner pregnancy policy, I am not sure honestly speaking I am not sure, but what I see in the clinic we have a register that we use to keep statistics of all pregnant learners we see.’ (Participant 5, female, CHN)

‘I don’t have much information on that to be honest [*about the learner pregnancy policy*]; I only know that in maternity there is a form that needs to be filled in by the parents of the child if the child is less than 15 years if the child is coming in for birth, that’s it.’ (Participant 2, male, CHN)

‘I am not sure if there is any [*learner pregnancy policy*] and it’s a problem because this thing of allowing them to go to school while pregnant I feel it’s also promoting this problem.’ (Participant 9, female, CHN)

Community health nurses’ lack of knowledge was a significant challenge because all the stakeholders involved in learner pregnancy management should be aware of the contents of the policy, particularly community health nurses and the DOH. With regard to training and availability of learners’ reproductive health programmes in the clinic, community health nurses revealed that no training or workshops had been provided. However, they indicated that there were clinic-based programmes that targeted teenagers. They were involved in youth programmes that focused on peer education but had never attended any training or workshop provided by the clinic. One youth programme that was commonly mentioned by community health nurses was the ‘Love Life’ programme, which focused on youth empowerment and addressed reproductive health issues:

‘I haven’t attended specific training per se, but I had a little background of peer education programme from university so I am incorporating it with my profession. I am also involved in a youth programme that has been established here in our facility to health educate learners, it’s actually a peer educator programme.’ (Participant 2, male, CHN)

‘There has never been any training…the management is also supportive isn’t now we have this programme of adolescents and it is very active and it is helping a lot because now learners are coming in numbers.’ (Participant 1, female, CHN)

‘I have never attended a workshop…but I am trained in this programme of youth-friendly services.’ (Participant 3, female, CHN)

Community health nurses revealed that there were youth-friendly services in clinics that targeted teenagers. These services targeted learners as well so that they could be encouraged to attend clinics. The purpose of youth-friendly services in the clinic was to have the reproductive health unit open for a longer period so that it could accommodate learners who came late from school. Community health nurses also allowed learners to come early in the morning before going to school so that they could receive first preference in the reproductive health unit:

‘Another thing is to inform learners about programmes that are available in the facility like youth-friendly services which are also here.’ (Participant 3, female, CHN)

‘Normally learners they do come late because of school and I always advise them to come earlier so that they can come in the morning so that they don’t become affected by this.’ (Participant 9, female, CHN)

‘We give them information on how they can cope with that pregnancy and antenatal visit on their flexible times because sometimes they will be writing their exams and they won’t be able to come for their appointment so we accommodate them.’ (Participant 4, female, CHN)

Clinics were playing a role in the prevention of pregnancy and provision of health education; however, the youth programmes and youth-friendly services in the clinic were not formalised.

### Theme 3: Stakeholders involved in learners’ reproductive health issues

Community health nurses emphasised the importance of stakeholder collaboration in addressing learners’ reproductive health. They agreed that if all relevant stakeholders worked together, learners’ reproductive health will be promoted. They identified the DOH as one of the stakeholders who play a major role in this issue. Other stakeholders that were mentioned included parents, teachers, nurses, the community workers and social workers:

‘I think parents, teachers and we as nurses and especially teachers who teach Life Orientation should also play their role, they should advise them that when you are sexual active…and parents at home should be frank with their kids.’ (Participant 8, female, CHN)

‘It was planned that we should go out to schools and raise awareness. Everyone, from the parents, the youth themselves and social workers, including the community as well.’ (Participant 2, male, CHN)

‘All of us, starting from home with parents, school educators, learners, the health system (Department of Health) and the sisters.’ (Participant 4, female, CHN)

School health nurses from the DOH were also highlighted as key role players in implementing reproductive health policies in schools. They further suggested that such policies should target all the identified stakeholders. The DOH’s role as the custodian of health is very significant in promoting school learners’ general health. Community health nurses suggested that the DOH ‘needs to do more’ because it was not doing enough to promote learners’ reproductive health:

‘People tend to forget that Department of Health has to work with more of those departments such as Department of Education and Social Services.’ (Participant 2, male, CHN)

‘I think the Department of Health is not doing much for me, what I see is that they are not doing what is supposed to be done.’ (Participant 10, female, CHN)

Stakeholder collaboration in promoting learners’ reproductive health was highlighted as an important part of policy initiatives. Community health nurses suggested that learner-centred policies such as the learner pregnancy policy and the ISHP should target important stakeholders such as parents, teachers, learners, nurses and social workers. The DOH and DOBE remain the custodians of implementing health promotion policies and must enable a multi-stakeholder approach in promoting learners’ reproductive health in schools.

## Discussion

This study aimed to explore the roles of community health nurses in the promotion of learner’s reproductive health in schools of Madibeng municipality, North West province. This study revealed that community health nurses were not visiting schools to provide health education and promote school learners’ reproductive health. However, such services were readily available at the clinic through youth-friendly services that targeted teenagers. The DOBE has introduced measures to address reproduction health education, such as the Sexual Education Programme and the Life Orientation subject (Maxwell, Radzilani-Makatu & Takalani 2016:1). Both these programmes include workshops for teachers so that they can be prepared to teach learners about reproductive health (Maxwell et al. 2016:1). Similar school health programmes were initiated by the Department of Education more than two decades ago. This includes the National Policy on HIV and AIDS for Learners and Educators in Public Schools and Student Educators in further Education and Training Institutions, and the 2003 National School Health Policy (Beksinska, Pillay & Smit 2014:676). These programmes have been revised and renewed over the years to cater for new challenges and cover areas that were initially omitted. However, it is still a challenge to involve community health nurses in schools through such school policy initiatives. This study suggests that such policy-based programmes should also include the role of community health nurses because reproductive health education is a health component.

The 2012 ISHP was introduced to renew and reinforce the National School Health Policy, and the general aim of the ISHP was to provide a comprehensive, integrated school health programme that is in line with the primary healthcare (PHC) package (UNESCO 2012). Moreover, Rasesemola, Matshoge and Ramukumba (2019:4) highlighted that the aim of the ISHP is to improve the physical, mental and general well-being of learners, which includes promotive, preventative and curative health initiatives. As much as the ISHP promotes a comprehensive approach, it falls short on providing guidance for the provision of reproductive health services in schools. Guidance on reproductive health services is vague because the current promotive, preventative and curative efforts focus only on school learners’ vision, hearing, mental health, and cognitive and developmental impairment issues (DOH & DOBE 2012:30).

The DOBE’s 2018 draft of the National Policy on Prevention and Management of Learner Pregnancy in Schools was developed to guide school officials, school management, principals, teachers and nurses (DOBE 2018:6). The policy seeks to address learner pregnancy by creating an environment with access to preventative measures and information that will encourage teenagers to make informed decisions concerning their reproductive health (DOBE 2018:6). When interrogating the implementation, use and accessibility of the learner pregnancy policy, Segalo (2020:4) found that teachers had access to the learner pregnancy policy; however, the policy could not be applied effectively because of its lack of specificity and practical ways of dealing with the problem in the school environment. This is supported by this study’s findings, which suggest that community health nurses do not visit schools to support teachers in dealing with reproductive health issues such as learner pregnancy in the school environment. Furthermore, this study indicated that there is no referral and follow-up care between schools and clinics regarding learner pregnancies. Further criticism about the learner pregnancy policy revealed that the policy is discriminatory as it only targets females and excludes male learners, who are often exonerated from taking responsibility for the pregnancy (Mndende 2021; Segalo 2020:4). A report by the Human Rights Watch (2018) stated that countries like South Africa that have learner pregnancy policies in place often lack implementation and monitoring plan, which is the most crucial part of any policy’s success. This study revealed that the role of key stakeholders is imperative in the implementation of reproductive health policies in schools, such stakeholders include community health nurses, parents, teachers, DOH and social workers. The DOBE should have a plan that provides specific guidelines that will ensure that the policy is respected, interpreted and fully used by school officials, teachers and education staff (Human Rights Watch 2018). Policy-related school-based interventions should be the effort of all stakeholders, including community health nurses, to raise awareness about learners’ sexual and reproductive health, as well as prevent teenage pregnancies in schools (Jonas 2021).

Community health nurses were not visiting schools, training or providing workshops to teachers regarding learners’ reproductive health issues; however, they are still needed in schools to work closely with teachers to address learners’ reproductive health. For learner pregnancy prevention initiatives to be effective, schools have to acknowledge and recognise the link between health and education, which essentially refers to the link between nurses and teachers (Mohlabi, Van Aswegen & Mokoena 2010:250). The link could be restricted by the DOH not doing enough to collaborate with the DOBE regarding the promotion of reproductive health of learners in schools. However, the collaboration between the DOH, DOBE and Department of Social Development was introduced through the 2012 ISHP (Beksinska et al. 2014). Netshikweta, Olaniyi and Tshitangano (2018:321) argued that addressing teenagers’ reproductive health problems is a global concern that should be alleviated by reproductive health programmes, which target important stakeholders such as parents, teachers and community health nurses. Whitehead (2016:270) also agreed that community health nurses as school stakeholders need to prioritise supporting schools, especially with the delivery of health education and other healthcare initiatives. Reproductive health education in schools has been encouraged by parents, especially because parents find it difficult to hold conversation containing reproductive health content with their children at home, attributing this barrier to cultural connotations (Netshikweta et al. 2018:320). This emphasises the important role community health nurses need to play outside the clinics’ vicinity, in order to reach out to learners and deliver reproductive health education in schools.

Community health nurses are currently providing preventative services in clinics, such as the services offered in the family planning unit. However, this is done without acknowledging the need to partner with teachers and schools to combat learners’ reproductive health problems. This study found that, even though community health nurses were not visiting clinics, they seemed to be far more welcoming of learners in the clinic premises. This finding was supported by youth programmes and youth-friendly services that were reported to be offered in clinics. Nonetheless, the youth-friendly programmes were not always effective as Ramalepa, Ramukumba and Masala-Chokwe (2020:37) found that school learners were not always willing to visit the clinic because of the negative attitudes of community health nurses towards them, especially in the reproductive health unit. This is supported by Beksinska et al. (2014) who also found that school learners were reluctant to visit government clinics regarding reproductive health issues, and this was attributed to the lack of training of healthcare workers regarding teenagers’ youth-friendly services in clinics.

## Conclusion

The study was justified by its aim, which was to explore the roles of community health nurses in the promotion of learners’ reproductive health in schools. Community health nurses were aware of their perceived role in promoting learners’ reproductive health in schools. They revealed that they were currently not involved in school visits; however, they suggested that nurses in the reproductive health unit were the ones catering to school learners when they visit the clinic. They further revealed that clinics were promoting youth-friendly services so that learners could come in numbers for reproductive health services. Stakeholders such as parents, nurses, teachers, the community workers, social workers and the DOH were identified as major role players in promoting school learners’ reproductive health. Community health nurses should view the school health promotion initiative as an opportunity to embrace the evolving broad-based health promotion concepts and seek to supplement health education by moving beyond the restrictions of providing services within their clinic facilities (Whitehead 2016:271). In an effort to promote a multi-stakeholder approach to health promotion in schools, the DOH and DOBE must reinforce the policy environment by safeguarding the implementation process of school health policies. Custodians of community health and education such as community health nurses and teachers need to have access to utilise and implement reproductive health-promoting policies in schools.

## Limitations

This report reveals the views of community health nurses and excludes the views of other relevant participants who were included in the main study such as learners, parents and school teachers. This limits the author to reveal an overview of the phenomenon of interest from all participants. The findings revealed by other participants were published in other reports.
